# Hospital and Institutional News

**Published:** 1912-03-02

**Authors:** 


### HOSPITAL AND INSTITUTIONAL NEWS.

### WORKMEN'S CONTRIBUTIONS TO VOLUNTARY HOSPITALS.

The question of the effect which the passing of the National Insurance Act is likely to have upon the contributions from the working classes to voluntary hospitals, and the view which these classes are likely to take in the matter, are of the gravest importance. Already the working men through existing organisations have the matter under consideration, and we have received a letter from the chairman of the Employees' Council in connection with the Birkenhead Borough Hospital enclosing the following resolution passed by them at a special meeting, when the National Insurance Act and its bearings upon the voluntary hospitals was fully discussed. Resolved: "That this meeting, which represents a very large proportion of the working men of Birkenhead, who contributed last year £1,440 4s. 5d. towards the total income of £4,416 11s. 11d. received by the hospital, is firmly convinced that when the National Insurance Act comes into operation there will be a very serious falling off in the workmen's contributions, and earnestly urges that Parliament will see its way to pass an amending Act, providing that out of the insurance fund such grant may be made to voluntary hospitals as they may need for their due support on their present basis; and that copies of this resolution be sent to Sir Henry C. Burdett, K.C.B., and Alfred Bigland, Esq., M.P."

### HOSPITAL INCOMES AND IMMEDIATE ACTION.

We recommend with some confidence the wisdom of the voluntary hospitals in each large city or town appointing a joint committee to represent the whole of them, and to instruct this committee to place itself in communication with the Employees' Councils, Hospital Saturday Fund Committees, and the representatives of the working classes in their district which support the voluntary hospitals. Then it should be relatively easy to thresh out the matter in all its bearings. At present there is time to prepare for combined action; but to sit still and do nothing until the crisis arises is a course which we feel pretty confident must result in a multiplication of difficulties, with the consequent risks of avoidable losses of income to the voluntary hospitals. At any rate the questions involved are gravely serious, and we hope many may send us their views so that every point may be considered on its merits.

### CROYDON'S NEW MEDICAL OFFICER.

Dr. M. B. Arnold, Senior Assistant Medical Officer of Health in Manchester, has been appointed Medical Officer of Health for Croydon. He succeeds Dr. Meredith Richards, now an Insurance Commissioner for Wales. There were 60 applicants for the post. Dr. Arnold is an M.D. of Manchester University, and has been engaged in much public health work. He has held the posts of House Physician to the Manchester Royal Infirmary and Senior House Physician to the City of London Hospital for Diseases of the Chest. He has also contributed many articles to the medical Press.

### HOSPITALS AND THE COAL STRIKE.

It is too early yet to state correctly the position of hospitals in the event of an industrial coal struggle. All that can be said is that the first institution to announce precautions is the Royal Infirmary at Derby. At Monday's meeting of the weekly board it was stated that the hospital possessed only three weeks' coal supply. The Board also apparently has given notice that in the event of difficulties occurring in the supply of food the number of in-patients may be reduced by dismissing all who are in a reasonably advanced condition to be moved.

### SIR THOMAS CROSBY ON THE COAL CRISIS.

In his capacity of Lord Mayor of London, Sir Thomas Crosby, M.D., summoned last Saturday a special meeting of Mayors to consider the situation threatened by the coal strike. Sir Thomas Crosby was extremely direct in his views. In dismissing the meeting he said: "We must be determined that the threatened serious interference with our fuel and our food supplies, our means of locomotion, and our industries shall not take place." The hospital world, and particularly the hospital world in London, will welcome this expression of opinion from the first Lord Mayor who is conversant with their needs in this matter.

### THE COLLIERS AND MANSFIELD HOSPITAL.

Mr. J. P. Houfton has been elected President of the Mansfield Hospital. This appointment, as Mr. T. W. Turner pointed out on the day of election, has significance from the fact that Mr. Houfton has been busying himself in inducing new subscriptions to the extension fund, for which, as we recently explained, a second £5,000 is needed. In this connection many will feel the weight of Mr. G. H. White's distinction between the generous sums as a rule contributed by the colliers and the sometimes grudging subscriptions which the companies remit. As Mr. White pointed out, to give an instance, if the Stanton Ironworks Company's workmen can, and do, give £320, the company can, and should, subscribe more than £20. Mr. H. E. Hollins, who was in the chair at the annual meeting, also pressed this point, though less categorically than Mr. White was inspired to do. We hope that the newly appointed committee, Mr. W. F. Jolly, Chairman of the House Committee, and Mr. Worman, its new member, will press home their responsibility upon the mineowners from the hospital's standpoint. The present coal crisis, however, seems unlikely to provide an opportunity of pressing any claims but their own upon the disputing parties.

### THE IRISH HOSPITALS CAMPAIGN.

The following signatures, representative of practically all the hospitals of Dublin, have been attached to a special appeal to protect Irish institutions from the disaster with which the Insurance Act threatens them: Marcus Goodbody, J.P., Adam Lloyd-Blood, Louis A. Byrne, M.D., Michael O'Dea, J.P.,

William Fry, J.P., H. Adair Hall, Colonel, Henry Jellett, M.D., John William Moore, M.D., Kt., John Marshall Day, M.D., Hon. Sec., members of Executive Committee. It is argued by these representatives, who have circularised the Irish newspapers, that the fact of "medical benefit" not applying to Ireland will jeopardise still further the position which their present threatened loss of subscriptions creates. This is a point worth making in Ireland, no doubt, but English hospital secretaries are under no illusion as to the benefits they may expect from an unamended Act, in which hospital benefit has not been incorporated. In any case the Dublin hospitals are justified in declaring their belief that as many out-patients as ever will throng their doors. Their solidarity in combining to show the disaster this invites is a praiseworthy example to English institutions, which, save for the fine efforts of the British Hospitals Association, were not quick at combined action on similar lines.

### THE AFTER-CARE OF IN-PATIENTS.

Only last week our Bureau drew attention to the problem of after-care for hospital patients, and alluded to the Northcote Trust's work for out-patients at St. Thomas's. In the interval the new report of that hospital's Samaritan Fund illustrates the question from another side. A most interesting statement is furnished by Mr. J. G. Wainwright, the treasurer, who, after reminding the public that this fund is now in its sixtieth year, sums up the work under three heads. The first is typified by the case of a child whose spinal complaint was remedied by a prolonged convalescence of ten and a half months at the Victoria Home, Margate, thanks to the Samaritan Fund, which enabled her to return to school. Another side of the work deals similarly with the relief of middle-class patients who have come down in the world, needing perhaps not merely operative treatment and convalescence, but also employment at their recovery. A third side shows how on a home being broken up by death and illness a whole family may be relieved. The interesting point about these examples is that it shows that a Samaritan Fund need not, and at St. Thomas's, at all events, does not confine itself exclusively to out-patients and their necessities. Another interesting fact is the keen group of workers which this side of hospital life attracts. Thus Mr. Peter Reid, the founder of the Swanley Home, the Baroness d'Erlanger, and Mrs. Sauer, of Harpenden, are the human pillars of much of this reconstructive work. The report is well worth reading, and the secretary of the Samaritan Fund, Mr. Sydney Phillips, deserves to be supported by everyone who cares for one of the most valuable departments of a modern hospital.

### THE TELEPHONE AND ADMINISTRATION AT BANSTEAD.

Feeling that the present domestic telephone system at Banstead Mental Hospital is of too limited a character to be compatible with efficient administration, and in order to establish direct and ready communication which is an essential for this purpose, the Mental Hospitals Committee of the London County Council propose that telephones to all the wards and detached buildings shall be provided. To do this a seventy-five line telephone exchange switchboard is to be installed in the hall porter's office; seventeen new telephone instruments will be required, and arrangements will be made for twenty-four instruments for future extensions. The estimated cost of this valuable improvement is £250. All of us have heard the tired superintendent retort on the admiring visitor who remarks, "You can speak to everyone here," that the reverse is the case, and that the increased efficiency of administration claimed for the telephone system is obtained by making the superintendent the slave of everyone under his control.

### A LISTER MEMORIAL FOR GLASGOW.

Nothing has yet leaked out as to the nature of the memorial which Glasgow has decided to erect to Lord Lister, beyond the fact that it will probably be on a large scale. A committee at present is considering various suggestions, and the result of their deliberations must be awaited.

### VISCOUNTESS PORTMAN AT THE SAMARITAN HOSPITAL, MARYLEBONE.

For some time past the accommodation for nurses at this hospital has been insufficient; and part of the nursing staff has had to be boarded out. This deficiency has been the subject of careful consideration by the governors, and the solution has been sought in the new nurses' home lately in course of erection on a site to the immediate west of the hospital, where some old property has been demolished to make way for it. The opening ceremony was performed on February 28 by Viscountess Portman in the presence of a gathering of subscribers, governors, staff, and others interested in the work of the hospital. Previous to the actual opening there was an exhibition in the hospital by the Ladies' Work Association. In the evening the committee of management and the medical staff of the hospital dined together at the Imperial Restaurant, an excellent custom which has long proved a success at various hospitals which have tried it. The new home is of red brick, and is designed in close conformity with the general architecture of the hospital itself; so that from the Marylebone Road the two buildings present the appearance of one harmonious whole.

### THE NEW NURSES' HOME.

The home is lofty, but not of any great width. There is a semi-basement, consisting of a fairly light front room, intended for a sewing-room; and of a very dark larger room behind, where the pathological museum of the hospital will be located. Above this is a suite of rooms for the matron, who is moving thither from the hospital itself, where the space thus gained will be allotted to the house surgeon. On the four upper floors are twelve more rooms, with bath and lavatory accommodation. Six of these are to be for the night sister and nurses-

Two more are nurses' sitting-rooms, and the large room hitherto devoted to this purpose in the hospital will be turned into a new ward. The rest of the home will be used as required to supplement the existing accommodation for nurses over the out-patient department. Each room is provided with an open grate, and is of a size compatible with the health and comfort of the occupant. The rooms are not yet furnished, but if specifications are followed such as some of those which have lately been illustrated in The Hospital, there should be little difficulty in making the new home as good as any of its kind in London.

### ALTERATIONS AT COLNEY HATCH MENTAL HOSPITAL.

The Mental Hospitals Committee of the London County Council have decided to spend £4,000 on the improvement of certain wards of Colney Hatch Mental Hospital to bring them up to the standard of modern requirements, especially as regards lighting and ventilation and the plastering of walls. We are informed that it is intended to improve three of the female wards and four of the male wards. The estimate of £4,000 is £1,000 more than the annual expenditure which was originally contemplated for this purpose, but the improvement of all the seven wards is very urgently needed, and in the committee's opinion none of them ought to be delayed for another twelve months. Also it is not possible to deal partially with any one block of buildings, as during the alterations it is necessary to remove the patients to other quarters. Nowadays the reaction from the old system is still remembered, with the satisfactory result that, if anything, a higher standard is demanded in mental hospitals than in the general run of institutions.

### THE PUBLIC AND HOSPITAL HISTORIES.

All who realise the value of hospital histories to the institutions concerned, and the interest of the subject itself, will have read with regret Mr. G. C. Peachey's recent letter in the *St. George's Hospital Gazette*, in which he records the small public which appears to have cared to possess the history of this hospital. Of course it must be admitted that the publication piecemeal, in parts, of any work tells against its success, but this is not quite enough to account for the want of support complained of. Again, hospital histories, like similar compilations, are sometimes hindered by delays. The history of St. Bartholomew's Hospital, if we are not mistaken, is an instance in point, and probably the success achieved by Mr. Morris's history of the London Hospital was almost as much due to its popular size, price, and treatment as to the undoubted literary ability and charm of style of its author.

### SOME NOTABLE WORKERS IN CUMBERLAND.

We are glad to see that the latest report of the Whitehaven and West Cumberland Infirmary refers markedly to the work of the late Mr. J. Gibson Dees, who had been chairman for seven years. When Mr. Edward Atter, the then vice-chairman, was appointed later to succeed him, Mr. Atter had already served for twenty-two years upon the committee. Mr. Atter's successor again, Mr. G. J. Muriel, had been for twenty-nine years visiting surgeon; so it is apparent that this hospital attracts steady workers. The County King Edward Memorial Fund produced rather over £500, which, we gather from Mr. W. H. Sands, the secretary, is to be used for a nurses' home, an addition long needed, and perhaps to be built on a site adjoining the Infirmary, which it is hoped to purchase. The fund, we understand, is still open, and we hope the locality will show continued interest in the project.

### HORSE AMBULANCES AND COUNTRY HOSPITALS.

Dr. M. R. Gooding took advantage of a formal speech he had to make to press the claims of a horse-ambulance upon his audience at Bideford. He instanced a recent accident which suffered from want of a proper form of conveyance to the hospital. The idea is now being taken up, the Mayor of Bideford, Mr. W. T. Goaman, and Capt. J. F. Stuart, R.N., having given a lead, and we expect that Mr. J. J. Lamerton will be able shortly to announce a sufficient sum for the purpose. If we may judge from last year's figures the hospital is well supported, and now that local generosity has renovated the theatre, no doubt this proposed adjunct to the theatre will soon be obtained.

### THE NIGHT BELL IN PRIVATE WARDS.

The problem of how best to summon the night nurse without unduly disturbing the rest of the ward is one that is not easily solved. At present most hospitals favour the electric-push bell. This, however, has its drawbacks. It is apt to get out of order, and refuse to ring or " indicate," where indicators are used, at the critical moment. If it rings it generally makes more noise than is desirable. Above all, it cannot always be easily manipulated by the patient, especially if it is a fixed bell attached to the wall. Where it is a pendant bell it can be more easily manipulated, but even then it needs a certain amount of energy on the part of the patient to use it efficiently. The method of electric lights now universal in American private wards, and recently introduced in Continental hospitals, seems to have several advantages. By lightly touching a button, conveniently placed within reach, the patient turns on the current which illuminates a numbered disc in the night nurse's room, the disc giving the number of the room or bed as the case may be. The light continues until the nurse switches off the current, so that there is no possibility of overlooking the call as sometimes happens with the bell. This arrangement is entirely noiseless, works smoothly and easily, and is simple in the extreme. It is at least worth a trial in our hospitals where separate rooms are provided for private patients.

### THE PERQUISITES OF CLUB PRACTICE

One of the points in connection with the stand which the medical profession is making against the provisions of the Insurance Act is not by any means an easy one for the general public to understand.

This is the argument of the doctors that six shillings per head per annum is too small a capitation fee. The public know that the current rates for club practice vary from about three-and-sixpence to five shillings for adults; and that four shillings is, broadly speaking, the usual payment. They also know that at these rates there is usually plenty of competition among members of the medical profession to secure the appointments. Hence they fail to understand how the doctors feel themselves aggrieved by the Chancellor's suggested rate. There are, however, several considerations which are not obvious to the casual observer, unacquainted with the inwardness of club practice. For one thing, the ordinary club consists of adult males, carefully selected as being healthy and generally earning good wages. The club doctor, if he is popular, reasonably expects a great many midwifery fees and an extensive practice among the large families which are the rule amongst the artisan class. The fees may not be large in themselves, but with an assistant a great number of patients can be seen in the day. This source of revenue to the club doctor will be a good deal interfered with by the Act.

### WHY DOCTORS UNDERTAKE IT.

Then again, all the members of a club do not call in the club doctor. Some may not like him, or at any rate, may prefer another practitioner, whom they consult and pay fees to. With free choice of doctor under the Act, these patients will be eliminated, and everyone for whom the capitation fee is paid will expect attendance. Others live too far away, and will not take the trouble to go to the doctor's surgery for minor ailments: these will probably send a peremptory message demanding a visit, or will be put on the list of some doctor in their own neighbourhood. Thus the present system really affords the club doctor something more than four shillings per member, to say nothing of the perquisites alluded to. But, over and above all this, it must not be forgotten that it is the poverty, not the will, of the medical profession that enables the club system to flourish. The prospect of a vast extension and of State countenance of this sweated labour has shown the profession that the best chance it has ever had of uniting for a living wage has arrived, and that it is a case of now or never. The perception of this dawned somewhat slowly upon the general practitioner; but now that he sees it plainly, not all the fury of the Chancellor, nor his intrigues with Sir Victor Horsley, are likely to make much impression upon the new-found solidarity of the rank-and-file of the profession.

### SOME STRENUOUS WORKERS AT CARDIFF.

The year 1911, especially to King Edward's Hospital, Cardiff, seems marked out for special mention in the history of that institution which one day will have to be written. The new system of patients' payments, which Mr. Leonard Rea described in The Hospital some weeks ago, was inaugurated in the course of it; the new wing was completed; the hospital was rechristened; a much-needed and most ably conducted appeal was launched successfully; the new theatre was opened, and, finally, it was Coronation year. This is surely enough to tempt the future historian to devote special treatment to it. As we go to press the report elaborating these facts is being submitted. If the meeting be made as interesting as the report—and Mr. Harry Johnson, of Leicester Infirmary, has given a telling example lately of how that may be assured—General Lee, chairman of the board of management, and his colleagues should deserve to hear that the debt on the year's work of about £1,000 has been paid by some supporter quick enough to grasp the importance of that work, and the really wonderful spirit which Col. Bruce Vaughan and Mr. Leonard Rea have infused into the work of this institution. The association of the hospital's appeal with the local memorial fund is another fact which those who like to understand the inner life of hospital history will recall with pleasure. The Board's report concludes with a reprint of The Hospital's article on Lieut.-Col. E. M. Bruce Vaughan, which in our "Eminent Chairmen" series appeared on July 29 last year. This article has proved to be not only a record of enthusiastic personal work but an interesting historical sketch of ten years in the life of this hospital.

### PATIENTS' CONTRIBUTIONS AT THE WEST END HOSPITAL.

If donations and subscriptions have been only fairly well maintained during 1911, and we learn that patients' contributions have substantially increased, Mr. D. D. Kirkaldy, the secretary of the West End Hospital in Welbeck Street, may congratulate himself that altogether ordinary income showed an increase over 1910 of £500, and if legacies were included this was doubled. A Ladies' Guild, as we have previously noted, has been established in aid of the hospital and a chapel also is being built free of cost, by the kindness of some friends of the institution. By the way, the hospital committee has lost the services of Mr. Welloch-Pollen and Mr. G. Huth, while on the other hand Lord Cardross, Mr. E. B. Hoare, and Mr. Cecil Higgins have been elected members. Dr. Palmer and Col. Willoughby responded to the votes of thanks passed to the medical staff and to the treasurer, at the annual meeting, when some slight amendments were made to rules. Among those present at it were Mrs. Ian Malcolm, Mr. A. Lister-Harrison, J.P., Mr. James Boyton, M.P., Mr. Leonard Brassey, M.P., Dr. F. S. Palmer, Dr. Harry Campbell (who was in the chair), Dr. Eric Macnamara, and Dr. Dundas Grant.

### BATLEY HOSPITAL AND ITS MEDICAL STAFF.

Mr. A. J. Riley, the newly elected president of the Batley and District Hospital, is already busy at work, a sub-committee having been appointed to consider a revision of the rules respecting the duration of the appointments of the honorary medical staff. Batley Hospital apparently has been in the habit of appointing its medical staff for an indefinite period, and so it may be conjectured that the sub-committee will recommend a limit of ten years or twenty, at the end of which the retiring medical men will be eligible for re-election to the consulting staff of the hospital. The new members of the committee are Mr. Dixon Oxley, whose father was one of the first life governors of the hospital, Mr. T. W. Liley, and Mr. G. H. Gale. Mr. A. W. Western is again secretary.

### ATTEMPTS AT A COUNTRY SCHOOL CLINIC.

However difficult the question of the treatment of children in the larger towns may be, it is still more difficult in country districts. In the majority of the counties the use of hospitals is scarcely a matter for consideration, since they contain virtually no hospitals which are readily accessible to the greater part of the population. Dr. Barwise, of Derbyshire, attempted to form a provincial club for the county on the basis of the present Insurance Act. There were ninety thousand children on the roll, and circulars were sent to the parents asking if they would be prepared to pay one penny a month or eightpence a year to insure for examination of their children's eyes and to provide spectacles, dentistry, the treatment of ears, adenoids, and tonsils. Forty per cent. of the parents proved to be able or willing to pay, but the committee did not consider the number to be sufficient to justify them in adopting the scheme. It was eventually decided, however, that such a club should be started for a limited area, which included two thousand children. The treatment, after consultation with the medical men in practice, it was arranged should be conducted by whole-time officers.

### SHOULD WHOLE-TIME SCHOOL OFFICERS BE LOCAL MEN?

Although treatment in school clinics conducted by whole-time officers has its dangers, it may be that the fears of some medical practitioners regarding the possibility of their being deprived of remunerative work by such officers are in a great degree groundless. The affections which are found in schools requiring treatment are of a nature that forms but a small part of the practice of the great majority of practitioners. Should, however, it not appear to be wise to appoint local practitioners to undertake the work of the treatment of school children, there seems to be no reason why the services of some practitioner outside the district should not be utilised, especially those of comparatively young men who have engaged in special work. A young medical man who has had experience of ophthalmic work would be glad of opportunities to maintain his skill in the somewhat difficult work of accurately testing defective eyesight. Presuming that the medical inspector who sends cases to the clinic has had some experience of children's diseases—which, in passing, it may be mentioned is by no means always the case—comparison of opinion between the inspector and an outside medical man of special experience could not fail to react favourably upon both.

### IS FIRST-AID TEACHING USELESS?

One member of the London County Council has suggested that the scheme of the Council's special classes for first aid, home nursing, hygiene, and infant care is not justified by the results. Mr. Cyril Cobb, Chairman of the Education Committee, however, quickly dispelled this idea. He stated that about 5,500 students took the various courses and had gone through the examinations, while about a third as many again took the courses but did not go in for the examinations. Of those who entered the examinations 77 per cent. passed. The total cost of the examinations was about £500, and the total cost of carrying on the whole of the classes would not amount to £5,000 a year.

### THE CHARING CROSS APPEAL.

Mr. Robin Duff was able to announce last week that the mortgage debt has been reduced by £21,000, a satisfactory, if not wholly successful, result of a year's work. It will come as no surprise that the closed wards are not to be opened till the debt has been further reduced, though we learn with interest that it is practically settled to open two wards for paying patients. The ordinary income has increased by approximately £1,500, which is a sign perhaps that the appeal is attracting new subscribers. Legacies still are apparently far to seek, and we can only hope that efforts to push forward the appeal may not be allowed to lessen at the present critical stage.

### AMUSEMENT AND MENTAL CASES.

Professor Davidson made good use of his opportunity at the annual meeting of Aberdeen Royal Mental Hospital, in emphasising two important points out of Dr. Reid's report. The first dealt with the number of admissions due to hereditary influences—no fewer than 30 per cent.—and is one aspect of the old enemy, recurrent insanity, only too well known to mental hospital workers. The second point insisted on by Professor Davidson was the proved value of intelligent amusement to mental cases. This, again, is, fortunately for all who have to enter mental hospitals, not now an innovation, but it is instructive to see how wide and varied a range such amusement may be made to cover. There is the cricket club mentioned approvingly by Professor Davidson. There is also the influence of the fine arts, particularly of music, which Dr. Theo Hyslop, for instance, initiated at Bethlem several years ago. Where we find such different interpretations of amusements as these two we may be sure that this form of treatment is being prosecuted in no narrow sense, and is getting its best results in consequence.

### CLUB CERTIFICATES FROM THE HOSPITALS' STANDPOINT.

When the Insurance Act gets into working order the certificates of ill-health demanded from hospital officers will doubtless be much more formal and precise documents than at present. As things are, each hospital provides a printed slip with spaces for the patient's name and illness, the date, and the resident medical officer's signature. These certificates are accepted without demur by the various clubs and benefit societies to which they are presented by their members. The filling up and signing of them are often very perfunctory affairs; for a busy house surgeon has little interest in protecting the pockets of wealthy friendly societies which he knows are always ready to sweat the profession. As matters stand, the certificates are at most hospitals issued gratuitously both to in-patients and out-patients. This in our judgment is a mistake, for many of the beneficiaries are well able to afford a small fee, and such fees in the aggregate would make a very appreciable source of hospital revenue.

### A HINT FOR HOSPITAL SECRETARIES.

It is, of course, not in accordance with voluntary hospital traditions that the bread-winner of a family, thrown out of work through illness, should be compelled to pay for a certificate out of the scanty sick-benefit upon which his family have to subsist until his recovery. But many working men have no dependants, and it is not uncommon for them to be receiving sick-pay from two or three clubs simultaneously; the resident medical officer finds this out when he is weekly requested to sign as many certificates. Such fortunate individuals are often able to provide themselves with grapes, bananas, and other delicacies out of their club-money; they seldom think of dropping anything into the hospital box in return for their treatment, and they should certainly be made to pay a shilling per certificate to the funds of the hospital. Resident medical officers should be requested to refrain from dealing out certificates *gratis*, but to refer all such demands either to the secretary's office or to the almoner. A system such as this would not only bring in revenue to the hospital, but would also remind the inmates of the wards and the frequenters of out-patient rooms that an elementary measure of gratitude is due to the charity which cares for them.

### THE DISINFECTION OF PATIENTS' CLOTHES

Considerable discussion has recently been going on in the German hospital Press regarding the best means of quickly and safely disinfecting patients' clothes. In the majority of cases the in-patients in Continental hospitals are not allowed to wear their own clothing; they must adopt the hospital uniform and even the underclothing and bed-clothing provided by the institution. Their own clothing in general is taken to the clothes room, which is usually in the basement of the administrative building, where it is disinfected in a Pfeiffer steam apparatus —a method in which formalin vapour is used in combination with high-pressure steam. This method is, however, expensive and is stated to be very damaging to the clothing. In the correspondence referred to some hospital authorities recommend disinfection by means of formalin vapour alone; this is efficacious but needs special cupboards or tinned cases. For the destruction of vermin in clothing carbon disulphide vapour is recommended, but where the clothes are much infested by vermin it is cheaper in the end to destroy them. In fact there is a consensus that in the case of pauper patients who arrive in rags, it is much more economical to burn the clothes and provide the patients with second-hand outfits, than to attempt disinfection. In English institutions the methods employed vary in nearly every hospital, but it would be interesting to have the experience of hospital managers on the subject. In some hospitals, we believe, there is no systematic disinfection of clothing at all, unless the patient is suspected of suffering from an infectious condition; the clothes are merely kept in a special room and handed back to the patient on his discharge. This is a careless system which should no longer be tolerated, since it introduces risks that no institution ought to be exposed to.

### WOMEN-DOCTORS IN GERMANY.

Some interesting figures about the medical profession in Germany have emerged from the statistics of the census of 1910. The total number is 32,449, not so very many more than the total for Great Britain if the estimates are correct upon which the contentions about the National Insurance Act are based. In Britain, however, the number of registered doctors increases nowadays very slowly, whereas in Germany it seems as if that increase is more rapid; so possibly in a few years the profession there will be as much overcrowded as it is here. Thus the number of medical students in Germany was nearly 2,000 greater in 1910 than in the preceding year. Women-physicians are also rapidly becoming more numerous; in 1908 there were only 55, in 1909 there were 69, and in 1910 there were 102—nearly twice as many as two years previously. Moreover the number of women-students is increasing by leaps and bounds, and bears a much larger proportion to that of the male students than in the case of the already qualified. Still, it cannot be said that Germany is over-doctored as yet.

### THIS WEEK'S DRUG MARKET.

Fluctuations in the values of drugs have not been numerous during the week, but the market in several articles, notably glycerine and cod-liver oil, is being watched with considerable interest. There is a possibility that the glycerine convention will be broken up; in which case a decline, probably a considerable decline, in value would be almost sure to take place. The present high price of refined glycerine is generally considered to be out of proportion to the cost of the crude article, and a little healthy competition would no doubt have the effect of reducing the price of the former; in any case, it would seem unwise to purchase, at present, more than is necessary for immediate use. Reports from the Norwegian fishing-grounds are very favourable, and the total yield of cod-liver oil is five times as large as it was at this time last year: it is further reported that the livers are yielding more oil than at the beginning of the season. It would therefore be wise to refrain from purchasing for the present. There is a slightly lower tendency in the price of carbolic acid, but this may not be maintained. Should the coal strike take place, the chemical industries will be very seriously affected and the prices of a number of articles would almost certainly be advanced. Camomiles are again dearer. Castor oil is slightly cheaper. The price of opium is well maintained, and there is nothing to indicate that a decline in value is probable in the near future. At the recent public sale of drugs in Mincing Lane full prices were paid for buchu leaves, gum benzoin, and sarsaparilla, but cascara sagrada and eucalyptus oil sold cheaply.

